# Green synthesis of silver nanoparticles via *Cynara scolymus* leaf extracts: The characterization, anticancer potential with photodynamic therapy in MCF7 cells

**DOI:** 10.1371/journal.pone.0216496

**Published:** 2019-06-20

**Authors:** Omer Erdogan, Muruvvet Abbak, Gülen Melike Demirbolat, Fatih Birtekocak, Mehran Aksel, Salih Pasa, Ozge Cevik

**Affiliations:** 1 Aydin Adnan Menderes University, School of Medicine, Department of Biochemistry, Aydin, Turkey; 2 Aydin Adnan Menderes University, Scientific Technology Research and Application Centre, Aydin, Turkey; 3 Sivas Cumhuriyet University, Faculty of Pharmacy, Department of Pharmaceutical Technology, Sivas, Turkey; 4 Aydin Adnan University, School of Medicine, Department of Biophysics, Aydin, Turkey; 5 Afyon Kocatepe University, Faculty of Education, Department of Science, Afyon, Turkey; VIT University, INDIA

## Abstract

In this study, we report on the synthesis of silver nanoparticles (AgNPs) from the leaf extracts of *Cynara scolymus* (Artichoke) using microwave irradiation and the evaluation of its anti-cancer potential with photodynamic therapy (PDT). Silver nanoparticles formation was characterized by scanning electron microscopy with energy dispersive x-ray spectroscopy and Fourier transform infrared (FTIR) spectroscopy. Silver nanoparticles formation was also investigated the surface charge, particle size and distribution using zetasizer analysis. The cytotoxic effect of AgNPs and/or PDT was studied by MTT assay and migration by the scratch assay. The apoptotic inducing ability of the AgNPs and/or PDT was investigated by intracellular ROS analysis, antioxidant enzyme levels (SOD, CAT, GPx and GSH), Hoechst staining and Bax/Bcl-2 analysis using western blotting. The mean particle size of produced AgNPs was found 98.47±2.04 nm with low polydispersity (0.301±0.033). Zeta potential values of AgNPs show -32.3± 0.8 mV. These results clearly indicate the successful formation of AgNPs for cellular uptake. Mitochondrial damage and intracellular ROS production were observed upon treatment with AgNPs (10μg/mL) and PDT (0.5 mJ/cm^2^) showed significant reducing cell migration, expression of Bax and suppression of Bcl-2. Significantly, biosynthesized AgNPs showed a broad-spectrum anti-cancer activity with PDT therapy and therefore represent promoting ROS generation by modulating mitochondrial apoptosis induction in MCF7 breast cancer cells.

## Introduction

In recent years, the interest in the synthesis and properties of noble metal nanoparticles such as gold, silver and platin has been attracting attention in nanomedicine [1]. Silver nanoparticles are widely used because of their unique properties and promising applications as anticancer and antimicrobial agents [2–4]. Three different synthesis methods have been developed for nanoparticle synthesis: physical, chemical and green synthesis [5, 6]. Physical methods require costly equipment, high temperature and high pressure. In the synthesis of nanoparticles with chemical methods, toxic chemicals are used which can cause serious damage to the environment and to the livings. Due to these disadvantages, the use of physical and chemical methods is limited. These methods are replaced by green synthesis which is a more environmentally friendly and cheaper method. Plants, bacteria, fungi, algae, etc. are widely used for the green synthesis of nanoparticles [7–9]. Many researchers reported the plant based green synthesis of silver nanoparticles using extracts of different plant parts such as peel, leaf, root, stem and fruit as natural resources [10, 11]. Various bioactive molecules found in these extracts, including proteins/enzymes, amino acids, polysaccharides, polyphenols, aldehydes and ketones that can reduce metal ions and stabilize the nanoparticles to desired shapes and sizes [12–14].

*Cynara scolymus* is a variety of a species of thistle cultivated as a portion of food. Artichoke extracts are known to exhibit anti-oxidant, anti-inflammatory, anti-allergic, anti-ulcerogenic and anti-hepatocellular carcinoma activity [15–17]. It has been reported that Artichoke extracts have a rich content of metabolites such as chlorogenic acid, luteolin, apigenin, cynarine, caffeic acid derivatives and flavonoids [18–22]. In this study, we have utilized the *Cynara scolymus* leaf extract (Artichoke) to synthesis bio- and eco-friendly AgNPs with green chemistry.

Photodynamic therapy (PDT) is a noninvasive therapeutic modality that based on the activation of a light-absorbing molecule called photosensitizer (PS) with light irradiation at a specific wavelength and generates reactive oxygen species (ROS) to the damage cancer cells. Compared with traditional therapy methods, the PDT technique has significant therapeutic efficiency and low side effects [23–25]. However, some of the factors, including the light used in PDT which can penetrate tissue, aggregation of the hydrophobic photosensitizer (PS) in aqueous media and the inefficient biodistribution of PS, reported as the limits of PDT. Therefore, an efficient drug delivery system is one of the challenges in this modality in order to overcome to these limitations. Ideally, the delivery system should be biocompatible and provide biodegradable of PSs in the target cells with minimized uptake by normal cells [26]. This study was designed to investigate the anti-cancer potential of well-characterized AgNPs and PDT combination therapy against breast cancer cells.

## Materials and methods

### The preparation of *Cynara scolymus* leaf extract

*Cynara scolymus* grown in the Aydın region were purchased in the local public market, two in each of three markets, (37°51'06.7"N 27°48'33"E; 37°51'1.9"N 27°50'42"E; 37°51'12"N 27°43'3.2"E) and were originated from Turkey. Each plant was purchased from May to July 2018. To permit identification of individual plants, each plant was labeled and defined organoleptic characterization as taste, color, odor, and feel. *Cynara scolymus* leaves were removed using scissors with freshly at the first day. The leaves of the *Cynara scolymus* were removed and washed 3 times with deionized water. Leaves were passed through the kitchen robot to separate small pieces (Arcelik, K 1190, Turkey). 200 g *Cynara Scolymus* leaves and 400 mL deionized water was added to 1 liter erlenmeyer. The mixture was heated in a magnetic heater (IKA, C-MAG HS-7, Germany) at 100°C for 2 hours. The mixture was filtered through Whatman filter paper (Grade 1) to give an extract.

### The synthesis of silver oxide nanoparticles

**Fig 1:**
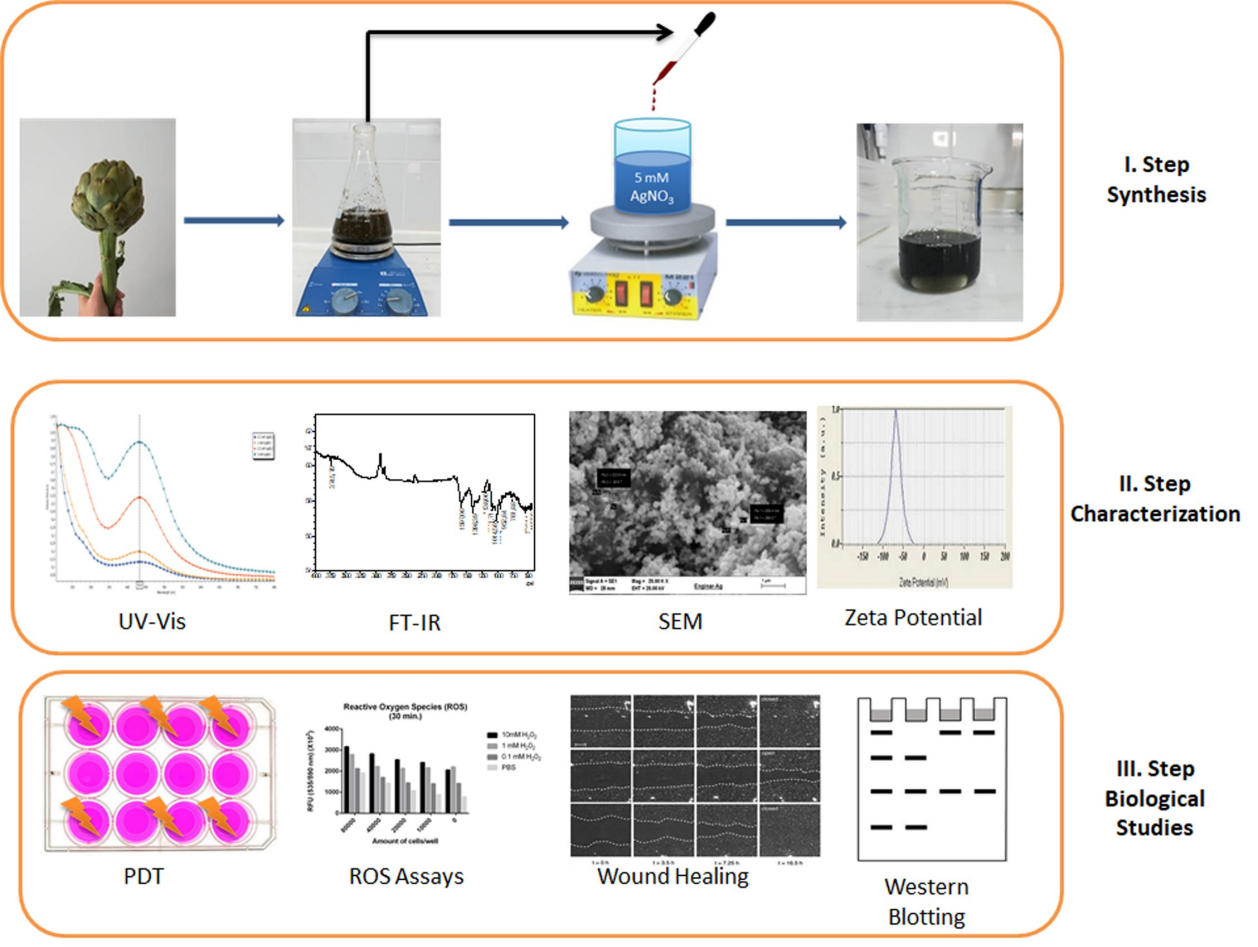
Schematic representation of nanoparticle synthesis with green chemistry.

20 mL of silver nitrate (10 mM) solution was added in a 100 mL beaker. 20 mL of *Cynara scolymus* extract was added dropwise to this mixture. It was placed in the ultrasonic bath for 30 min. Then it was subjected to 360 W microwave (Vestel MD-20 MB, Turkey) irradiation for 5 min. The mixture was centrifuged for 20 min at 4000 rpm. To remove organic residues, the pellet was washed 5 times with ethanol. The powder form of nanoparticles obtained by lyophilization and was kept at room temperature for further study (Fig 1).

### Characterization of silver oxide nanoparticles

The formation of Ag nanoparticles was observed [27] by Ultraviolet-visible (UV-Vis) spectrophotometer (Thermo Scientific Multiscan Spectrum 1500, USA) with a range of 200–800 nm. The functional groups on AgNPs were validated with Fourier-transform infrared (FTIR) spectroscopy using in the range of 400–4000 cm^-1^(Shimadzu IR 8000, Japan). The surface morphology and the structural properties of AgNPs were characterized by SEM (LEO 1430 VP, Germany). Elemental compositions were analyzed by EDX (LEO 1430 VP). Particle size, distribution and zeta potentials of nanoparticles were determined using Zeta Sizer (Nano ZS-90 Malvern Instruments, England). An aliquot of nanoparticles was diluted with pure water, then sonicated for 10 min before the measurements.

### Cell culture

MCF7 breast cancer cells (ATCC HTB22, Rockville, MD, USA) were cultured and maintained in DMEM (Gibco, Invitrogen Australia) containing 10% FBS (F2442, Sigma, USA) and supplemented with 100 U/mL^−1^ penicillin and 100 μg/mL streptomycin (Gibco). The cultures were incubated at 37°C in a humidified atmosphere with 5% CO_2_. All experiments were repeated multiple times, representative results shown.

#### PDT treatment for cell line

For experiments, the cells were irradiated with 0.5 mJ/cm^2^ light intensity of the light from a solar simulator (Model 11018 Abet Technologies, USA) and midOpt SP785-105 infrared dichroic block filter and LP590-105 red longpass filter. AgNPs were incubated 10 μg/mL concentration during 4 hours in MCF7 cells and irriadiated with PDT. After the treatment, the cells were cultured for 24 hours and then prepared for various analyses.

#### Antiproliferative assay

The effects of AgNPs with/without PDT combination on MCF7 breast cancer cell viability were determined [28] using a tetrazolium-based microplate assay with MTT (Vybrant, Life Technologies). Briefly, The MCF7 cells were seeded into a 96-well plate at a density of 1×10^4^ cells/well. After incubating the cells for 24 h, the dilutions of AgNPs at different doses (0.1–1000 μg/mL) were added and incubated for 24 h, 48 h and 72 h. After that, the culture medium was discarded and the wells were washed with PBS twice, followed by the addition of 20 μL MTT dye (0.5 mg/mL) each well. The cells were incubated for another 4 h at 37°C. After removing all the culture medium, 150 μL DMSO was added per well. The percentage of cell viability was measured on ELISA reader (Biotek Co., USA) at wavelength of 490 nm. The cell inhibitory rate was calculated using the Graphpad Prism 7.0 programme.

#### Crystal violet staining

AgNPs with/without PDT treated cells were cultured, fixed with absolute ethanol for 20 seconds, stained with 0.4% crystal violet (Sigma C0775, USA) for 5–10 min, later on washed with PBS until clear and images were taken by using the inverted microscope. For cell counting, 51% of Triton X 100 was added and absorbance was measured at 470 nm by ELISA reader (Biotek Co., USA).

#### Hoechst 33342/PI double staining

Propidium iodide (PI) and Hoechst 33342 double staining [27, 29] in cultured MCF7 cells were measured to detect apoptosis after the treatment of AgNPs with PDT. Cells (1 × 10^5^) were cultured in a six-well plate and treated with different concentrations of AgNPs then eight hours incubated with PDT exposed cells. Control cells were maintained without adding AgNPs. After a 24-h incubation, cells were washed with PBS and then fixed with 70% ice-cold ethanol for 10 min. The fixed cells were washed with PBS and stained with Hoechst 33342 (1 μg/mL, Sigma 14533, USA) and propidium iodide (5 μg/mL, Sigma P4170, USA) for 10 min. After discard of the excess dye by washing with PBS repeatedly, images of cells were captured under a fluorescence microscope (Olympus BX51, Japan).

### ROS measurement

Quantitative measurements of Reactive Oxygen Species (ROS) were determined cytofluorimetrically using the MUSE Cell Analyzer with Oxidative Stress kit (Millipore, Billerica, MA, USA) according to the manufacturer’s protocol. MCF7 cells were grown on 6 well plates. After the treatment AgNPs with/without PDT, the cells were washed twice with PBS and then incubated with ROS reagent. 10 μL of cell suspension in 1X Muse assay buffer was added to 190 μL of working solution reagent. The samples were mixed and then incubated for 30 min at 37°C, subsequently read by Cell Analyzer (MUSE Cell Analyzer, Millipore, Germany).

#### SOD, CAT and GPX activities assay

Superoxide Dismutase (SOD), Catalase (CAT) and Glutathione Peroxidase (GPx) enzyme activities of MCF7 cells lysate were measured with commercial activity assay kit. SOD activity was measured the dismutation of superoxide radicals generated by xanthine oxidase and hypoxanthine using manufacturer's protocol assay kit (Cayman 706002, Canada). CAT activity was measured for the detoxification of hydrogen peroxide (H_2_O_2_), with 4-amino-3-hydrazino-5-mercapto-1,2,4-triazole (Purpald) as the chromogen using manufacturer's protocol assay kit (Cayman 707002, Canada). GPX activity was measured with glutathione reductase (GR) reduced state by NADPH using manufacturer's protocol assay kit (Cayman 703102, Canada). All experiments repeated multiple times, representative results shown.

### Bax and Bcl-2 protein expression with western blotting

After treatment of AgNPs for 24 h, total cellular proteins were prepared using RIPA lysis buffer (20–188 Merck Millipore, Germany) (included protease inhibitor cocktail) from MCF7 cells [30]. The protein concentrations were established by bicinchoninic acid assay (71285-Merck Millipore, Germany). Equal amount of protein was separated by 12% polyacrylamide gels and then transferred onto PVDF membranes (sc-3723, Santa Cruz, USA). The membranes were blocked with 2.5% BSA at 4°C overnight and then incubated with specific primary antibodies (Bax (sc-7480) and Bcl-2 (sc-7382), Santa Cruz, USA). After washing with TBST (containing 0.1% Tween 20) 3 times, the membranes were incubated with the corresponding HRP-conjugated secondary antibodies in TBST at 37°C for 1 h. The protein β-actin (sc-47778) was used as a housekeeping control for normalization. Finally, the expression levels of proteins were visualized and analyzed using ImageJ software.

### Wound healing assay

Cells (1x10^5^ cells/well) were seeded into a 12 well culture plate and were allowed to grow overnight to reach confluence in DMEM media [30]. Cells were treated with AgNPs during 4 hours and were exposed with PDT. The monolayer was then scratched with a pipette tip, washed with PBS twice to remove floating cells, and treated with control media. After incubation period of 24 h, the cells migrated into the scratched area were photographed under a phase-contrast inverted microscope. The distance that cells had migrated into the cell-free space was measured by Image J software. The width of each migrated area was used to calculate the relative proportion wounded at time zero.

### Caspase-3 activity assay

In order to determine the cells levels of caspase-3 activity, cells were lysed with cell lysis buffer and centrifuged for 10 min at 9000 rpm at 4°C after treatment of AgNPs and/or PDT for 24 h. Using a commercial kit (Calbiochem, USA) and following the manufacturer's instructions, the supernatant was used for measuring caspase-3 activity as a marker of apoptosis in cells.

## Results

### Characterization of synthesized AgNPs

**Fig 2:**
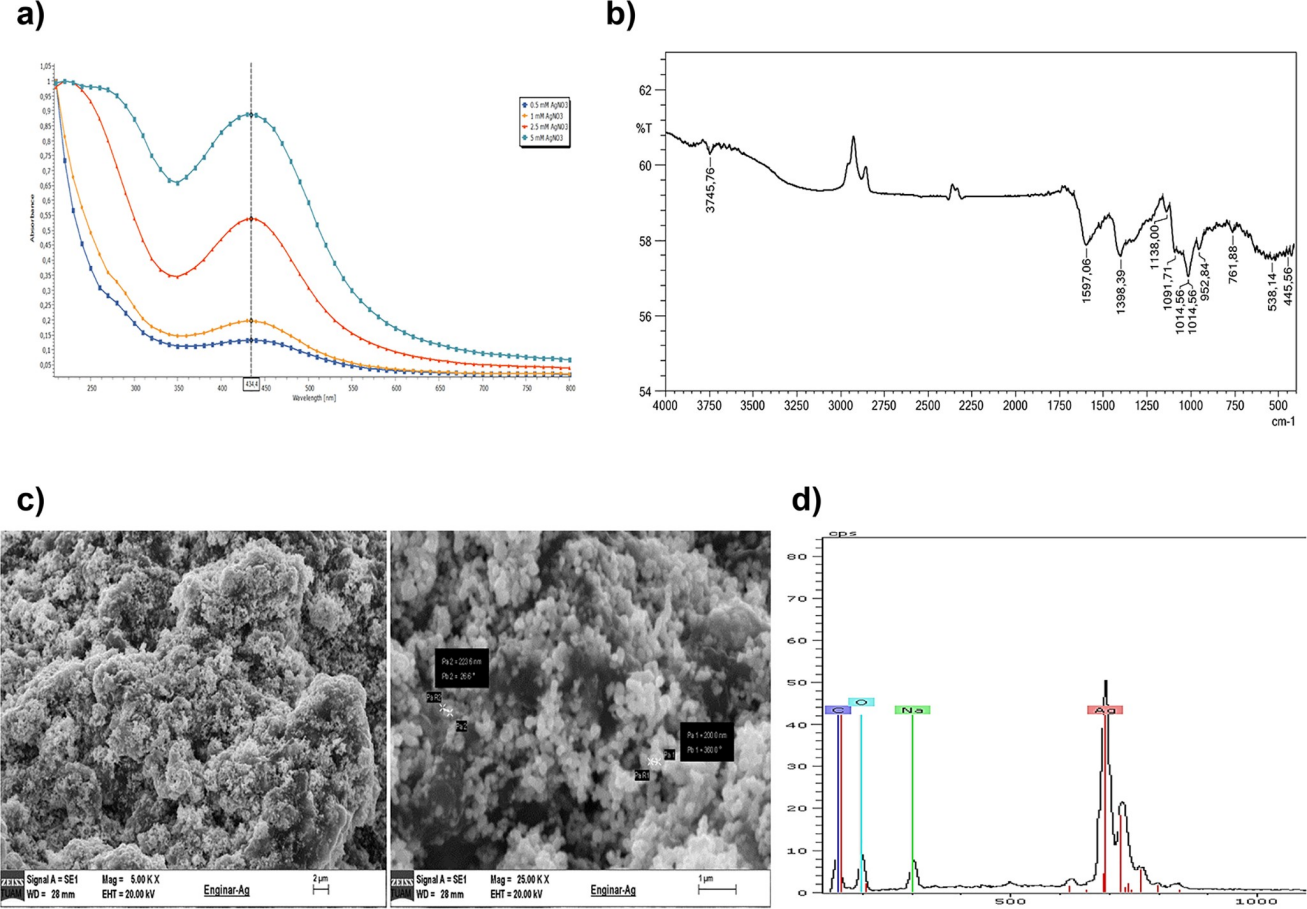
Characterization of AgNPs. **a)** UV spectrum, **b)**FT-IR spectrum, **c)**SEM images at different magnitudes and **d)** Energy-dispersive X-ray spectroscopy spectrum of AgNPs.

The changing color of the reaction mixture (visual observation) during the reaction time is the main indication of nanoparticles synthesis. This color change occurs due to the excitation of the surface plasmon on metal nanoparticles. The appearance of a brown-orange coloring is evidence of formation of the AgNPs. The characteristic peak around 400–450 nm is specific for AgNPs [31, 32]. Fig 2A shows UV- Vis spectra recorded from *Cynara scolymus* leaf extracts and different silver nitrate concentrations. The absorption spectrum of AgNPs spanned a wide range from 330 to 640 nm with a prominent peak at 434 nm (Fig 2A). This peak indicates the formation of AgNPs owing to the range of the surface plasmon resonance (SPR) for AgNPs [33].

Broad bands in FTIR spectrum demonstrate the existence of AgNPs [34, 35]. Stretching vibrations at 538 cm^-1^ can also be attributed to the reduction of Ag^+^ to Ag. The presence of different functional groups on the AgNPs, and their related bonding information is presented in Table 1. The disappearance of most functional groups such as carbonyl and hydroxyl may be caused due to the bioreduction during the occurrence of AgNPs (Fig 2B).

**Table 1:** Identification of the vibrations of the synthesized AgNPs.

FT-IR Analysis		
Wavenumber cm^-1^	Bond	Functional Group
1597	C-C stretch (in ring)	Aromatics
1398	-CH_2_- bending	Aliphatics
1138	C-N strech	Aromatic amines
1091	C-N strech	Aliphatic amines
1014	C-O strech	Ether

Scanning electron microscopy technique has been used to identify the morphology and size of the bioreduced AgNPs [36]. Fig 2C represents the SEM images of silver nanoparticles (Ag-NPs) synthesized using *Cynara scolymus*. The nanoparticles appear as aggregated and spread uniform shapes. Silver nanoparticles seem also spherical with 200–223 nm average diameters.

The elemental composition of powdered AgNPs was determined by using SEM equipped with an EDX detector. The EDX spectrum shows the existence of silver nanoparticles as 59.59% (Fig 2D). Other contaminants such as carbon, oxygen, and sodium probably were caused by *Cynara scolymus* residues.

Particle sizes of AgNPs were found 98.47±2.04 nm size with relatively homogenous distribution (polydispersity index: 0.301±0.033). The zeta potential is an indicator of surface charge potential which is an important parameter for understanding the stability of nanoparticles in aqueous suspensions. Zeta potential values of AgNPs were measured -32.3±0.8 mV. It was stated that produced nanoparticles had negatively charged on their surface.

### Cell proliferation with AgNPs and/or PDT therapy

**Fig 3:**
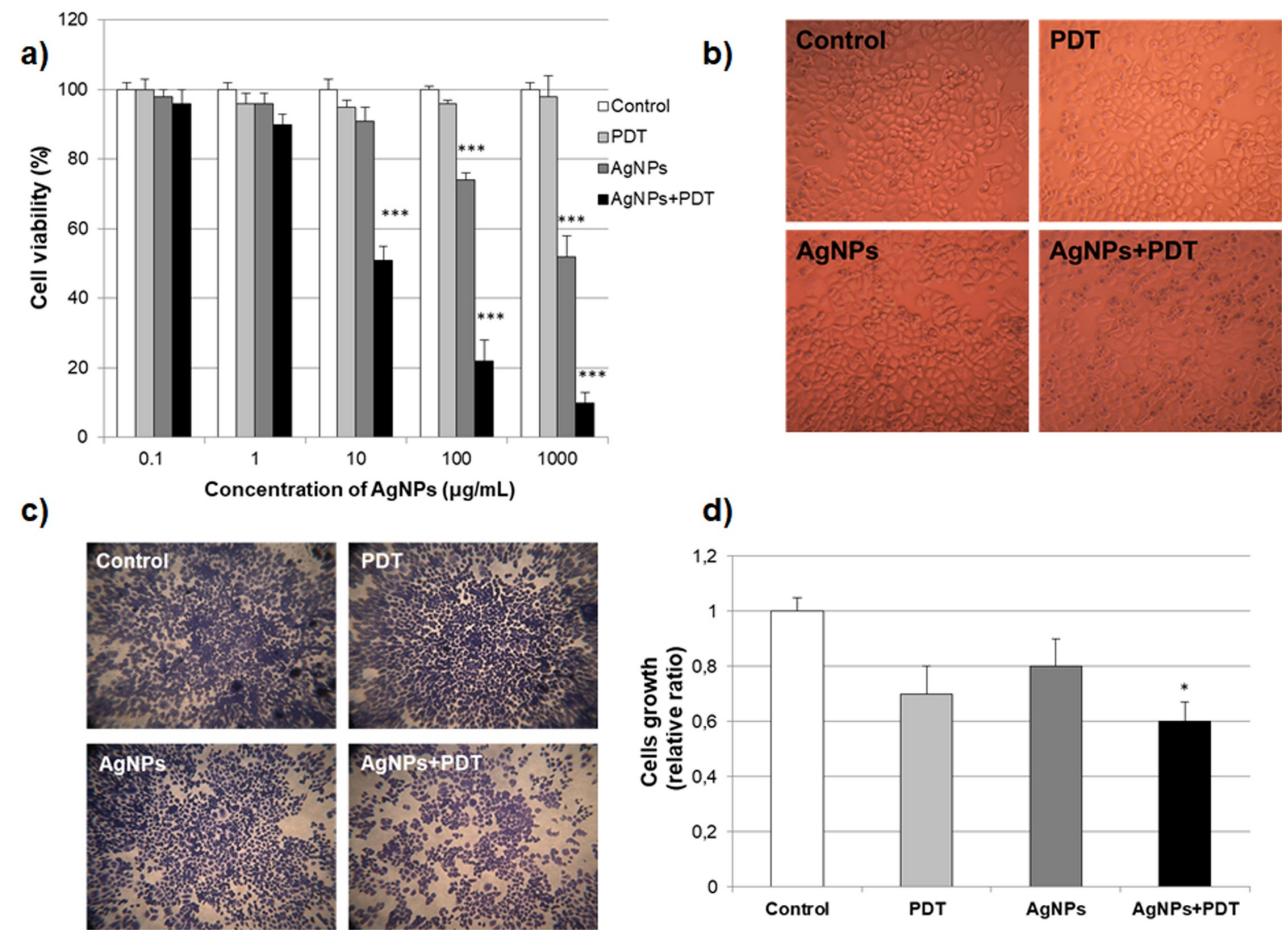
Effect of AgNPs and PDT therapy on the cell survival in MCF7 breast cancer cells. **a)** Cell viability measurement with MTT assay after AgNPs and/or PDT treatment (0–500 10 μg/mL) of MCF7 cell lines for 24 h. **b)** Cell morphological changes after treatment AgNPs and/or PDT treatment MCF7 cell lines for 24 h. **c)** Cell growing stained with crystal violet **d)** Ratio of cell growing in MCF7 cells with treated of AgNPs (10 μg/mL) and/or PDT for 24h (*p<0.05 compare to control) for the three biological replicates within each group.

MCF7 breast cancer cells were exposed to AgNPs at the concentrations of 10 μg/mL and/or PDT for 24 hours, and cytotoxicity was determined using MTT assay (Fig 3A). Results have shown that AgNPs down to the concentration of 100 μg/mL did not produce a significant reduction in viability of MCF7 cells. Only 10 μg/mL concentration of AgNPs has not changed cell viability although cell viability was decreased with combination of AgNPs (10 μg/mL) and PDT therapy (Fig 3A and 3B). Combined treatment with 10 μg/mL AgNPs+PDT therapy in the MCF7 cells suppressed cell proliferation after 24h (Fig 3C and 3D). Since the total number of cells was reduced after 24h treatment with 10 μg/mL AgNPs and PDT combination, we performed cell morphological changes and crystal violet staining.

### Oxidant/Antioxidant status with AgNPS and/or PDT therapy

**Fig 4:**
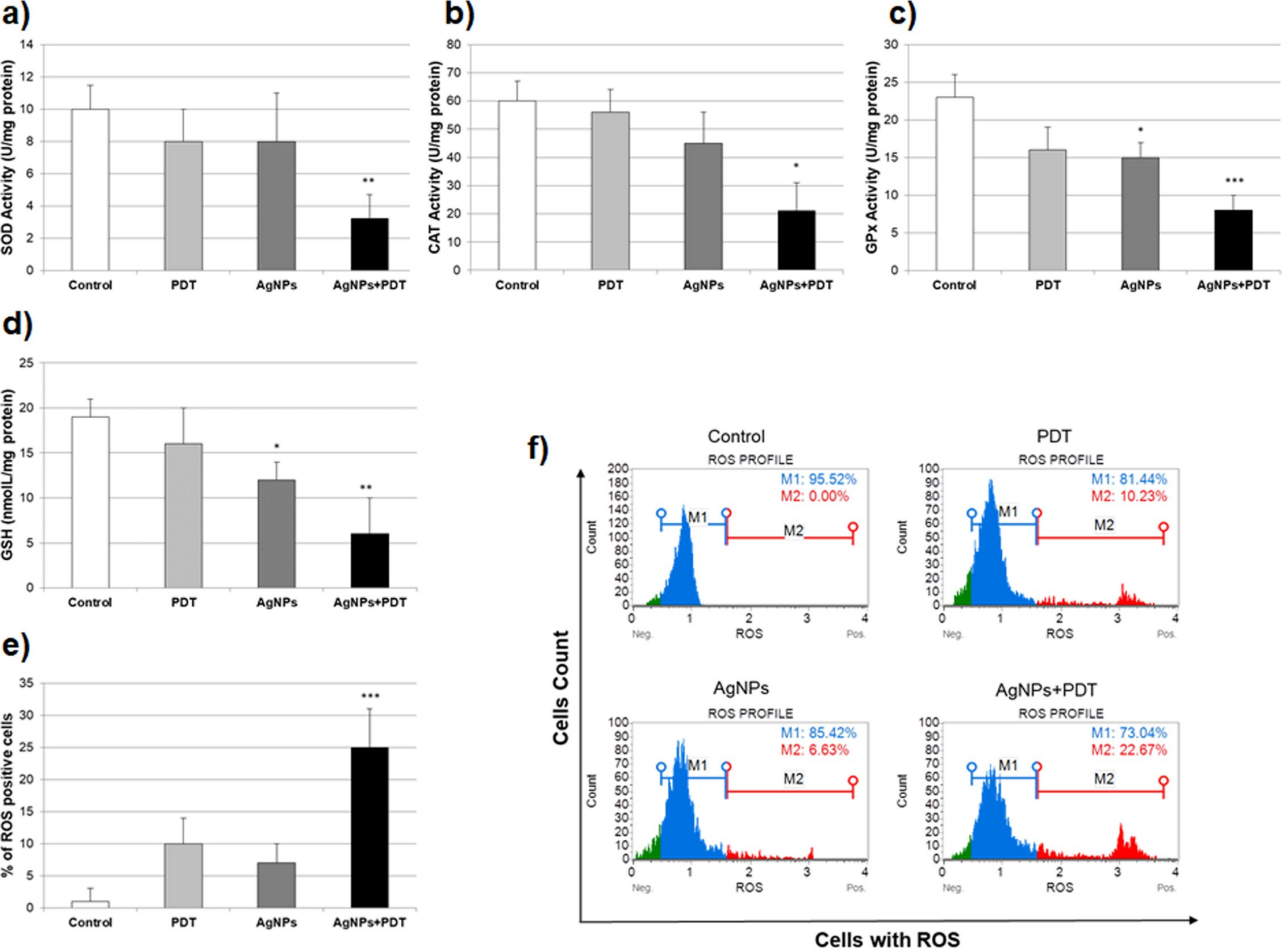
ROS generation following incubation of the MCF7 cells with AgNPs (10 μg/mL) and/or PDT for 24h. **a)**SOD activity **b)**CAT activity **c)**GPx activity **d)**GSH levels **e-f)**Percentage of ROS generation plots on Muse Cell Analyzer (*p<0.05, **p<0.01, ***p<0.001 compare to control).

To investigate the effect on the oxidant/antioxidant status, MCF7 cells were treated with 10 μg/mL AgNPs and/or PDT for 24h and then antioxidant enzymes were measured with enzymatically by CAT, GPx, SOD and GSH. Combination treatment with AgNPs and PDT decreased SOD and CAT activity by diminishing production of free radicals in MCF7 cell line. AgNPs or PDT therapy were not induced the oxidative stress via SOD and CAT activity (Fig 4A and 4B). AgNPs caused a significant decrease in GPx activity when compared to control. Compared to the untreated control, treated AgNPs+PDT induced a significant decrease GPx levels in the generation of reactive oxygen radicals (Fig 4C). Similarly, AgNPs and AgNPs+PDT caused a significant decrease in GSH levels when compared to control (Fig 4D). Cellular total ROS production levels were measured muse cell analyzer with treated AgNPs and/or PDT cells. The results showed that the cellular ROS productions were increased in AgNPs+PDT groups. Compared to the control group, in all groups that received ROS production, respectively, only statistically significant changes were observed in AgNPs+PDT groups, as shown in Fig 4E and 4F. This effect was observed releasing of reactive oxygen radicals production in AgNPs+PDT treated of cells.

### Cell migration with AgNPs and/or PDT therapy

**Fig 5:**
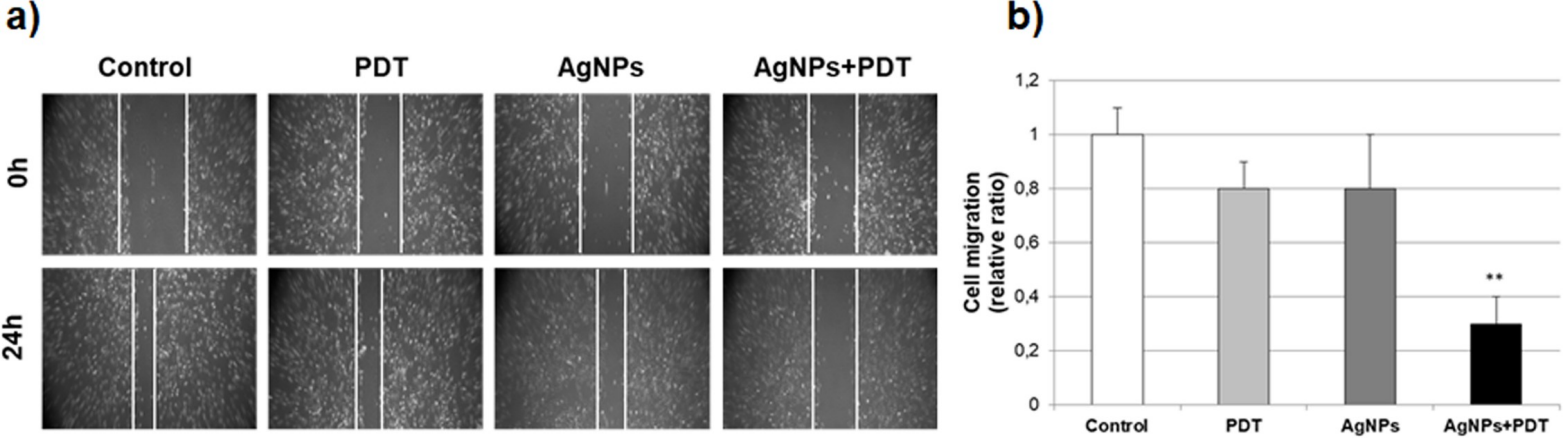
Effects of AgNPs and PDT therapy on cell migration in MCF7 breast cancer cells. **a)** Cell migration measurement with scratch wound assay in AgNPs (10 μg/mL) and/or PDT for 24h treated MCF7 cells. **b)** The rate of wound closure was calculated differences of cells filling the scratched area (**p<0.01 compare to control in MCF7 cells) for the three biological replicates within each group.

Cell migration ability was assessed using scratch wound-healing. In the scratch wound-healing assay, cells with the AgNPs or PDT alone groups filled the wound completely after 24 h, while the combination of AgNPs and PDT groups exhibited reduced migration into the wound in MCF7 cells for 24h (Fig 5A and 5B). The AgNPs+PDT group also exhibited a decrease in migration; however, the effect was less than that for the PDT alone group at the same light dose.

### Cell apoptosis with AgNPs and/or PDT therapy

**Fig 6:**
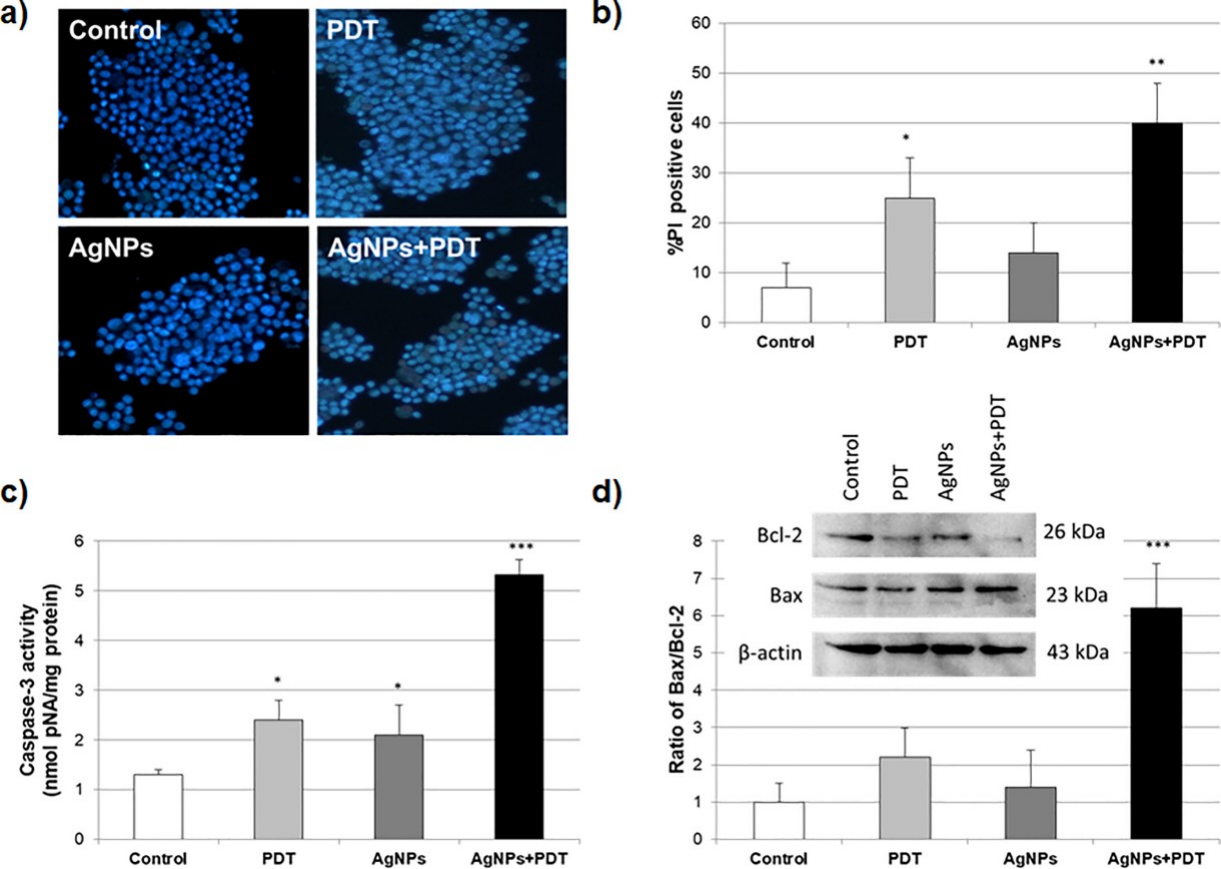
Effects of AgNPs and PDT therapy on apoptosis in MCF7 breast cancer cells. **a)** Hoechst 33342/PI Double Staining of AgNPs (10 μg/mL) and/or PDT for 24h treatment in MCF7 cells. **b)** Percentage of PI staining cells of AgNPs (10 μg/mL) and/or PDT for 24h treatment (*p<0.05, **p<0.01 compare to control). **c)** Caspase-3 activity of AgNPs and PDT therapy for 24h in MCF7 cells (*p<0.05, ***p<0.001 compare to control). **d)** Western blot bands of expression levels of Bax and Bcl-2 proteins in AgNPs (10 μg/mL) and/or PDT for 24h treatment in MCF7 cells. Each protein band was normalized to the intensity of β-actin used. Western blot densitometry analysis of ratio of Bax/Bcl-2 protein expression levels in MCF7 cells. (**p<0.01 compare to control cells) for the three biological replicates within each group.

To investigate the effects of AgNPs and/or PDT treatment on MCF7 cell apoptosis, these cells were treated with 10 μg/mL AgNPs and/or PDT for 24h and then analysis of apoptotic proteins and Hoechst 33342/PI staining were performed. The Hoechst 33342/PI staining experiments showed a significant increase (20% higher than the control level) in the PDT alone treated MCF7 cells. Moreover, within the AgNPs+PDT treated groups, the Hoechst 33342/PI staining level was elevated along with the increasing treatment AgNPs (Fig 6A), with statistical significance (Fig 6B). These results suggest that the combination of AgNPs and PDT treatment could trigger the apoptosis of MCF7 cells.

As an indicator of apoptosis, caspase-3 activity in the AgNPs and/or PDT for 24h was elevated in the AgNPs+PDT treated groups with respect to control group, while treatment with either AgNPs or PDT therapy enhanced the apoptotic activity in the MCF7 cells (Fig 6C). Western blotting was performed to evaluate the mitochondrial apoptosis expression levels of the Bax, Bcl-2 and GAPDH (control) proteins. Our results showed that photodynamic irradiation alone could not influence the mitochondrial apoptotic proteins as Bax and Bcl-2. On the other hand, without photodynamic irradiation, alone treatment AgNPs did not result in significant differences in the Bax and Bcl-2 protein expression rate. Importantly, the ratio of Bax/Bcl-2 protein expression of MCF7 cells treated with AgNPs+PDT was dramatically increased compared with controls (Fig 6D).

## Discussion

Silver nanoparticles are widely used in the drug carrier, cancer treatment, anti-microbial studies, nanotechnology, biotechnology and biomedical field. Over the past few decades, AgNPs synthesis strategies have focused on the development of eco-friendly and rapid green approach for cost effective synthesis methods [37–41]. Therefore, this reaction pathway satisfies all the conditions of a 100% green chemical process in which plants, fruit, bacteria, yeasts, fungi etc.are used [42, 43]. This study reports on the anti-cancer activities of AgNPs prepared from *Cynara scolymus* leaf extract samples, which have been green and cost effective synthetic method, the combination with PDT.

Particle size is an important indicator of product quality and performance. It is also crucial for nanoparticle accumulations in the tumor tissues through vascular gap of the tumor capillary which was reported to be in size up to 400–600 nm. This phenomenon, called enhanced permeability and retention (EPR) effect, provides higher accumulations of nanoparticles with smaller particle size than 400 nm into the tumor [44, 45]. Particle sizes of AgNPs were found 98.47±2.04 nm size with narrow distribution. With this green synthesis method, it was possible to obtain particle sizes lower than even 100 nm. It can contribute to higher drug internalization into the cancer cells. The zeta potential is an indicator of surface charge potential which is an important parameter for understanding the stability of nanoparticles in aqueous suspensions. The positive or negative charge on the surface of nanoparticles provides stability and prevents aggregation of nanoparticles by pushing the same charges [46]. Zeta potential values of AgNPs were measured -32.3± 0.8 mV. It has been stated in the literature that nanoparticles with a zeta potential higher than +30 mV or lower than −30 mV are considered to be very stable in the dispersion medium [47]. These results highlighted that AgNPs can preserve their structure over the long-term and clearly indicate the successful formation of AgNPs for cellular uptake.

PDT is defined as the use of a particular type of light via compatible photosensitizing agents such as drugs, nanoparticles or chemicals in the treatment of cancer [48]. PDT treatment causes cells death by apoptosis and/or necrosis with the generation of free radicals and singlet oxygen in cells [49, 50]. The combination treatment of PDT and green synthesized AgNPs leads to the increased production of intracellular ROS formation and decreased antioxidant status such as SOD, CAT, GPx and GSH in MCF7 cells. Furthermore, this combination inhibits the cell growth, cell viability and cell migration by 50% (IC50) which is about 10 mg/mL, via free radical generation in MCF7 cells. This finding is in accordance with previous reports for AgNPs and PDT treatment, suggesting that ROS generation changes the dynamic balance between oxidation and antioxidant levels by reducing Ag^+^ and AgNPs [51, 52]. In addition, AgNPs and PDT incorporation in MDA-MB-468 breast cancer cells increased by three times of intracellular ROS production comparing to control [53]. The production of ROS associated with mitochondrial phosphorylation is also involved in pro-apoptotic mitochondrial events in tumor cells processes which the cell undergoes apoptosis [54]. The generation of mitochondrial ROS response frequently includes an upregulation or activation of pro-apoptotic proteins and downregulation or inhibition of anti-apoptotic proteins in cell death [55]. The green synthesized AgNPs from *Cynara scolymus* leaf extract with PDT therapy, showed efficient anti-cancer activities via mitochondrial apoptosis in MCF7 cells. The intrinsic apoptosis pathway was induced by AgNPs and PDT combination therapy through up-regulation of pro-apoptotic proteins of the Bax and downregulation of the anti-apoptotic proteins Bcl-2 in MCF7 breast cancer cells. Interestingly, a previous study on the influence of silver nanoparticle suggested that AgNPs synthesized from *Rubus fairholmianus* extract induce mitochondrial apoptosis without PDT in MCF7 cells [56]. We have shown mechanistically AgNPs with PDT therapy activates the intrinsic apoptotic pathway in MCF-7 breast cancer cells. This eco-friendly synthesized AgNPs could be a competitive alternative to the conventional physical/chemical methods and have a potential to use in anti-cancer treatment with PDT therapy for breast cancer in the near future.
